# Designing ionic channels in novel carbons for electrochemical energy storage

**DOI:** 10.1093/nsr/nwz140

**Published:** 2019-09-13

**Authors:** Jianglin Ye, Patrice Simon, Yanwu Zhu

**Affiliations:** 1 Hefei National Research Center for Physical Sciences at the Microscale & CAS Key Laboratory of Materials for Energy Conversion & Department of Materials Science and Engineering, University of Science and Technology of China, Hefei 230026, China; 2 CIRIMAT UMR CNRS 5085, Université Paul Sabatier, Toulouse 31062, France; 3 Réseau sur le Stockage Electrochimique de l'Energie (RS2E), FR CNRS 3459, Amiens 80039, France; 4 iChEM, University of Science and Technology of China, Hefei 230026, China

**Keywords:** carbon materials, ionic channels, ion kinetics, electrochemical energy storage

## Abstract

Tremendous efforts have been dedicated to developing high-performance energy storage devices based on the micro- or nano-manipulation of novel carbon electrodes, as certain nanocarbons are perceived to have advantages such as high specific surface areas, superior electric conductivities, excellent mechanical properties and so on. In typical electrochemical electrodes, ions are intercalated/deintercalated into/from the bulk (for batteries) or adsorbed/desorbed on/from the surface (for electrochemical capacitors). Fast ionic transport, significantly determined by ionic channels in active electrodes or supporting materials, is a prerequisite for the efficient energy storage with carbons. In this report, we summarize recent design strategies for ionic channels in novel carbons and give comments on the promising features based on those carbons towards tailorable ionic channels.

## INTRODUCTION

**Figure 1.**
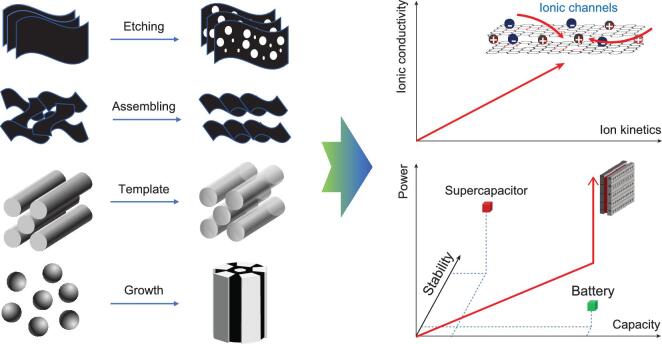
Typical methods of fabricating ionic channels with lengths across microscopic to macroscopic scales, and the corresponding aims for better energy storage.

Ions have been utilized for electrochemical energy storage in the last two centuries. Ion batteries rely on the reversible ionic intercalation/motion of Li^+^, Na^+^, K^+^, Zn^2+^ and so on [1–4]; electrochemical capacitors (ECs), on the other hand, store energy with ion adsorption (in electrochemical double-layer capacitors, EDLCs) or fast surface redox reactions (in pseudo-capacitors, PCs) [5]. Compared to electronic transport, ionic transport is slower yet complicated in electrodes. Charging/discharging processes in batteries typically take several hours for the efficient use of internal space or pores [6]; EDLCs may be fully charged/discharged within a few seconds if the ionic transport is fast enough [7]. Therefore, high ionic conductivity and optimized ion kinetics in electrodes are desirable for better electrochemical energy storage (Fig. 1).

More recently, *sp*^2^ hybridization-dominant carbon nanomaterials, including activated carbons, carbon nanotubes, graphene, and their derivatives have been widely investigated to enhance the energy storage performance with regulable structural and diverse properties, including—but not limited to—excellent electric conductivity, good mechanical and chemical durability [8–10]. Although these nanocarbons have shown potential for electrochemical energy storage under laboratory set-ups/conditions, charging/discharging processes are essentially limited by ionic kinetics/fluxes in the electrodes, which could be a big challenge under harsh working conditions (e.g. at temperatures lower than −30 C, or for rates higher than 10 C in batteries) [11]. The specific charging mechanism enables EDLCs to have remarkable power performances; however, the limited capacity (<10 Wh/kg) related to the migration of ions into active sites cannot yet meet requirements. Generally, the inner-pore ionic transport and the external ionic diffusion from bulk electrolyte to pores are the main factors influencing the ionic kinetics, and a significant electrode potential drop and a low ion accessible area may be inevitable when these two resistances are notable, especially for large currents, thus severely deteriorating the performance. When the thickness of carbon electrodes or areal mass loading is scaled up to practical levels (beyond 100 μm thickness or 10 mg/cm^2^ areal loading), the restriction from ion diffusion would be even more remarkable [12–14]. Ideally, efficient ionic channels in carbon electrodes should generate fast electrolyte transport by migration and diffusion, excellent accessibility of electrolyte in the inner pores, as well as fast cathodic/anodic reactions if the Faraday process is involved.

Ionic channels in different carbons shall be treated respective to different electrochemical processes. For instance, graphite, as the commercial anode for lithium (Li)-ion batteries (LIBs) [15,16], has shown a much lower capacity when being used as an anode for sodium (Na)-ion batteries (SIBs) due to the insufficient interlayer spacing and larger energy barrier for intercalation/extraction of Na^+^ [17,18]. In contrast, expanded graphite with larger interlayer spacing has exhibited better capability for Na^+^ storage [19]. Indeed, novel carbons with high specific surface areas (SSAs) and controllable ionic channels could be ideal candidates as electrodes for energy storage, by facilitating the ionic transfer and enhancing the accessible area in electrolytes. But the side reactions at the carbon/electrolyte interface shall be considered [20,21].

Here we present a brief summary of the proceedings in the design, fabrication and qualification of ionic channels in novel carbons for electrochemical energy storage. We begin this review with the fabrication strategies for ionic channels in carbons and the state-of-the-art of studies on energy storage applications, in which the relationship between ionic channels and the resultant performances will be highlighted. Then the ionic responses and dynamics among ionic channels, including the constitutive effects of size and surface chemistry, are presented. As a relatively straightforward model of 2D ionic channels, graphene stackings are discussed for the fundamental understanding of ionic adsorption/transport in the confined space. The review ends with a summary of unresolved challenges and an outlook on the topic.

### FABRICATION STRATEGIES FOR IONIC CHANNELS IN CARBON

In most situations, ionic channels originate from the porous structures in carbons, which may be fabricated with controllable processing protocols. The prevailing strategies for fabricating pores and the relevant products are summarized in Fig. 1. To date, attempts to fabricate the ionic channels in carbons mainly include [9,10,22–24]: (1) creating pores or defects on the surface or in the matrix with, e.g. physical or chemical activation/etching; (2) coupling/self-assembling multi-dimensional carbons with guest species to form hybrids; (3) tuning microstructures with the assistance of templates; (4) assembling the channels by the aggregation of nanoscale building blocks. The success of these approaches requires a deep understanding of the microstructure and the charging mechanism on how this in turn affects the energy storage. Generally, it has been accepted that micropores contribute to the ion adsorption, especially in EDLCs, whereas meso/macro-porous ion-buffering micro reservoirs facilitate the ion penetration or transportation from electrolyte to the inner surface of carbon-based electrodes [25,26]. In this case, the ion-transport time (τ) is a key to achieving fast ion kinetics, which is given by the equation τ = *L*^2^/*D*, where *L* is the ion-transport length and *D* is the ion-transport coefficient [27,28]. Micropores usually have a long diffusion distance, while mesoporous channels may provide nanometer-scale diffusion routes (e.g. <100 nm) [29]. Following this principle, a high utilization of micropores with short τ for supercapacitors may be achieved in interconnected hierarchical materials (broad pore size distribution (PSD)) due to the nanoscale transport distance from adjacent ionic channels, and increasing *D* in carbons is another efficient way to achieve fast ion kinetics in batteries.

**Figure 2.**
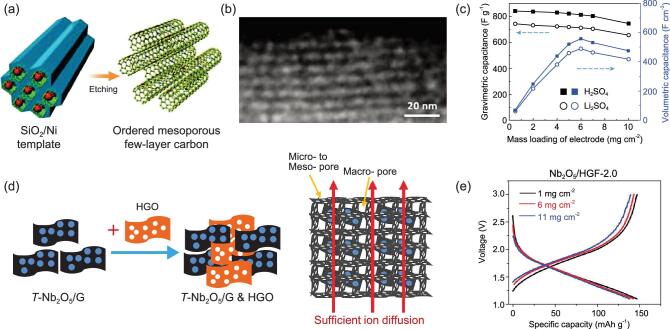
(a) Schematic of an ordered mesoporous few-layer carbon after the template is removed. (b) High-angle annular dark-field transmission electron microscopy (HAADF-TEM) images of the ordered mesoporous carbon, in which dark regions indicate connected ionic channels. (c) Gravimetric and volumetric capacitance (measured at 1 A/g) of a symmetric electrochemical cell device versus areal mass loading of the corresponding ordered mesoporous carbon electrode in aqueous electrolytes. Adapted with permission from [34], Copyright 2015, American Association for the Advancement of Science. (d) Illustration of the two-step process to prepare 3D hierarchically porous composite architecture with efficient ion diffusion (additional notes have been added in the composite architecture). (e) Galvanostatic charge–discharge curves for porous graphene/Nb_2_O_5_ with different mass loading. Adapted with permission from [40], Copyright 2017, American Association for the Advancement of Science.

Among the methods mentioned above, template assistance has been widely utilized to fabricate ionic channels [26,30], in which carbon microstructures and nanoarchitectures are directly formed from predefined microporous or mesoporous patterns [31]. The sizes and shapes of channel arrays can be tuned by changing the features of the template. So far, several templating materials, such as mesoporous silica, NaCl crystal, superlattices of Fe_3_O_4_ nanocrystals and so on, have been investigated to achieve regular arrays; and a variety of nanostructured templates, such as SiO_2_ hollow nanotubes, and pluronic nanospheres have been used to synthesize networks with well-aligned nanochannels [32,33]. Taking the fabrication of mesoporous carbons as a typical example, as shown in Fig. 2a, the use of mesoporous SiO_2_/Ni template resulted in the ordered mesoporous N-doped carbon as expected [34]. The highly ordered and homogeneous mesoporous channels (4–6 nm in width) were considered to facilitate the mass transport of ions and solvent to active N-sites on the inside wall. Furthermore, the porous tubes, containing uniform pore channels of 1 to 2 nm in diameter (Fig. 2b), minimized the solid-state diffusion path length in electrodes with a large thickness. As a result, this carbon showed an impressive gravimetric capacitance of 855 F/g in aqueous electrolyte, as seen from Fig. 2c. The excellent performance was maintained up to a mass loading of 8.0 mg/cm^2^, indicating the good access of electrolyte without significant restriction in diffusion. The capacitance retention was over 90% after 50 000 cycles, demonstrating the robust porous architecture. Nevertheless, challenges remain due to the instability of nanoscale templates under high temperatures or solvent treatments, and it is highly expected that such periodic carbon materials serve as the model platform to investigate the structure–property relationship.

More than providing the sites for ion storage and electric transport, carbon additives with highly interconnected networks can also increase the ionic transport/migration of active components [35,36], especially for high-rate battery electrodes (e.g. larger than 10 C) and/or a mass loading of >10 mg/cm^2^. For example, it has been reported that a 70-μm-thin LiNi_1/3_Mn_1/3_Co_1/3_O_2_ electrode showed a capacity of 131 mAh/g at C/2, which, however, dropped to 86 mAh/g for the thickness of 320 μm at the same rate [37]. In the carbon-hybridized electrodes, a more uniform distribution of potential facilitates the ion accessibility and reactions on active sites and regulates the transport of ions and solvent molecules [32], leading to improved mass transport in electrodes [38]. Various techniques, e.g. the hydrothermal process, capillary compression, layer-by-layer and electrostatic self-assembly, have been utilized to prepare the hybrids including conductive carbons and other active components [39]. An interesting example is that an electrode with 3D porous carbon frameworks (HGF) as the ion/electron-conducting scaffold and orthorhombic niobium oxide (Nb_2_O_5_) as the electrochemically active material has been developed, schematically shown in Fig. 2d [40]. The obtained 3D Nb_2_O_5_/HGF electrode showed an excellent rate capacity of 139 mAh/g at 10 C under a mass loading of 11 mg/cm^2^ (Fig. 2e), much higher than the typical high-rate graphite anode (∼80 mAh/g at 1 C) [41]. The stable cycling maintained for 10 000 cycles at 10 C with a Coulombic efficiency of above 99.9%, demonstrating the robustness of the ionic channels, which can be considered as a critical step forward towards practical applications of high-power LIBs. Other promising composites such as sulfur nanocrystals anchored on interconnected fibrous graphene and highly dispersive metals/metal oxides on carbons with designed ionic channels have also been reported [42–45], providing efficient mass transport for high-rate capacity.

As the perfect carbon shows low activity for the adsorption and reactions of ions to some extent, a wide range of studies have focused on the functionalization of *sp*^2^-hybridized carbons, e.g. by engineering defects, doping and so on, to significantly improve the capacity of supercapacitors [46,47] and batteries [48]. As an example, the pores in graphene sheets etched by H_2_O_2_ have sizes of 1–2 nm, comparable to or larger than those of organic electrolyte ions [49]. In contrast to wormlike tortuous pore channels, these in-plane defects may benefit the ionic transport between neighbor layers and maximize the accessible surface area. This holey graphene framework exhibited an impressive gravimetric capacitance of 200 F/g at a current density of 100 A/g in 1-ethyl-3-methylimidazolium tetrafluoroborate/acetonitrile (EMI BF_4_/ACN) electrolyte. On the other hand, heteroatom doping, typically substituted by B, N, S, and F atoms, has shown ability in the delicate controllability of electrical, chemical and other properties of novel carbons [50]. Among them, N atoms can increase the n-type carrier concentration and surface energy of carbons, improving the surface reactivity [51]. For example, controlled N-doping in carbon channels has been realized during the chemical vapor deposition (CVD) of ordered mesoporous carbon by introducing NH_3_ into the gas flow [34]. With ∼8.2 at.% N doping and higher wettability, lower interfacial diffusion resistance, the N-containing redox reaction can be bipolarly charged/discharged at a speed of several seconds (typically with a relaxation time of 2.1 s), resulting in a capacitance over 500 F/g. The advantages of highly active sites for electrochemical reactions and the effective ion accessible areas in N-doped carbons have also been utilized to improve the Na^+^ transport kinetics. In this regard, porous 2D or 3D N-doped carbons with open ionic channels are promising for high-performance SIB electrodes (e.g. for a capacity of 400 mAh/g at 0.1 A/g) [52,53]. Other studies have been devoted to improving the performance based on coupling effects, including using multiple heteroatoms and optimizing atomic structures [50,54]. However, controllable defects are still far from expectation.

### ION RESPONSES IN IONIC CHANNELS WITH SIZES, DEFECTS AND HETEROATOM EFFECTS

**Figure 3.**
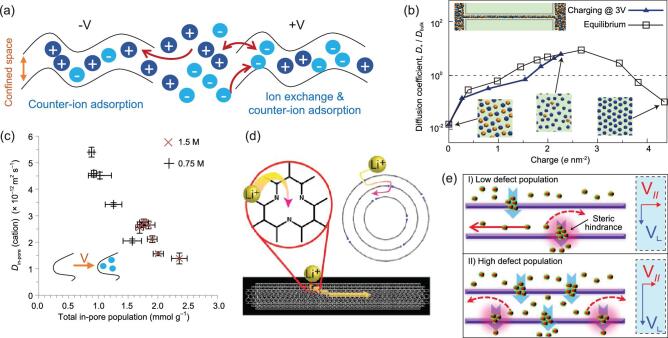
(a) Schematic showing a typical charge storage process in EMI TFSI electrolyte determined by EQCM experiments. (b) Diffusion coefficient of cations in a silt channel (width: 0.53 nm) along the equilibrium path and an impulsive charging at 3 V; the inset shows the molecular dynamics simulation featuring an electrode channel and part of the room-temperature ionic liquid reservoirs connected to it. Adapted with permission from [62], Copyright 2014, Springer Nature. (c) Correlation between the self-diffusion coefficient (*D_δ_*) of in-pore cations and total in-pore ion population for microporous carbon electrode (YP-50F) with tetraethylphosphonium tetrafluoroborate (PEt_4_ BF_4_) salt dissolved ACN as the electrolyte from *in situ* NMR experiments (the additional schematics show the increased *D_δ_* in initially empty pores). Adapted with permission from [59], Copyright 2017, Springer Nature. (d) A schematic illustration showing that defected nitrogen configurations in the wall of CNTs provide an efficient ion channel for lithium ion intercalation. Adapted with permission from [65], Copyright 2012, American Chemical Society. (e) Schematics of the proposed Li diffusion mechanism through defects on the basal plane with different defect populations. Adapted with permission from [66], Copyright 2012, American Chemical Society.

Despite the various processes above, the detailed mechanism governing the adsorption and diffusion of ions in a confined space is not yet completely understood; one desires to know how the pore/channel sizes and surface properties of the pores/channels affect the ionic behavior. In supercapacitors, first-principles simulation has indicated that nanochannels with sizes much larger than electrolyte ions (e.g. 1-nm pore versus 0.45-nm tetraethylammonium (TEA^+^)) in few-layer carbon nanosheets favor ion penetration/diffusion, while large ions (e.g. 0.8-nm tetrabutylammonium (TBA^+^)) show increased interaction with carbons on the edges of nanopores through chemical bonding [55]. *In situ* characterizations, e.g. nuclear magnetic resonance (NMR), electrochemical quartz crystal microbalance (EQCM) and scattering approaches, have shown that different charging mechanisms may occur depending on the electrode polarity when a potential is applied on the carbon electrode in EDLCs [56]. In an experiment on carbide-derived carbon (CDC, with a pore size of 1 nm) positively charged electrode with 1-ethyl-3-methylimidazolium bis (trifluoromethanesulfonyl)-imide (EMI TFSI) ionic liquid (IL) electrolyte, ion exchange was first detected at low charging; counter-ion adsorption then dominated at higher charging (Fig. 3a) [57]. At the negatively charged electrode, however, counter-ion adsorption seemed to dominate over the whole range of charge studied. In a CDC electrode with smaller nanopores (∼0.65 nm), no mass change on the quartz was observed upon cycling, meaning that the ions cannot access the ultra-micropores. Furthermore, when the IL was diluted with acetonitrile, the increased mass changes were confirmed in the negative electrode, which have been assigned to the solvent molecules (partial desolvation when accessing small nanopores) carried by EMI^+^. Similar phenomena of ionic electro-sorption using a microporous activated carbon electrode (YP-50F) were observed using *in situ* NMR and *in situ* NMR combined with EQCM [58]. On this basis it is inferred that ion-exchange and counter-ion adsorption processes may be the universal charging mechanisms in porous carbon electrodes [56]. However, other factors, e.g. ion sizes and in-pore ion populations, would bring more variations of in-pore ionic diffusion during charging as shown by the *in situ* NMR spectroscopy experiments on YP-50F, due to the increased ion–ion interactions in confined space (steric and electrostatic effects) [56,59]. Kaneko and

co-workers [60] found that monolayer IL confined inside carbon nanopores breaks its Coulombic ordering, leading to the formation of co-ions pairs and the increase of capacitance. Thus, under physical limitations, the charging mechanism shall relate to the ion–ion and ion–carbon interactions, and the motional rates of anions and cations [56], based on which the controlled energy storage may be realized by optimizing the sizes and surfaces of ionic channels and pores, as further discussed below.

**Figure 4.**
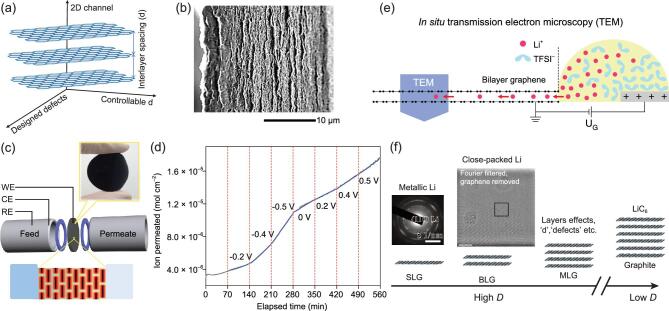
(a) 2D ionic channels among graphene stacking. (b) Typical SEM image of cross sections of a graphene film containing layer-like channels for ion storage and transport. Adapted with permission from [69], Copyright 2013, American Association for the Advancement of Science. (c) Schematic showing the experimental set-up for the investigation of ion diffusion through nanoconfined EDLs in charged, layered graphene-based nanoporous membranes, and (d) steady-state diffusion curves obtained under a programmed potential. Adapted with permission from [74], Copyright 2018, Springer Nature. (e) Schematic of an *in situ* transmission electron microscopy (TEM) device with bilayer graphene electrode [80], and (f) TEM image showing the metallic Li on SLG—adapted with permission from [81], Copyright 2019, Wiley-VCH—and multi-layered close-packed Li crystal forming inside BLG during lithiation from the *in situ* TEM result. Adapted with permission from [80], Copyright 2018, Springer Nature.

The surface chemistry, e.g. modification by doping or defect engineering mentioned above, would certainly affect the behaviors of ions compared to `smooth' carbon channels due to the distinct difference in electronegativity, surface energy and carrier concentration among defects. Also, taking N-doping as an example, improving the wettability by N modification is an efficient way to improve the electrochemical performance in EDLCs and PCs, while at the expense of conductivity to some extent [61]. Obviously, there could be a fine balance between the effect of overcrowded ions and that of electric conductivity. Both mean-field theories (MFT) and molecular dynamic (MD) simulations show that an ionophobic channel with single slit shape (0.53 nm) performs better in terms of charging rate in a room-temperature ionic liquid, leading to a large self-diffusion coefficient (*D_δ_*) of ∼2.57 × 10^−8^ m^2^/s (comparable to or higher than the bulk system, Fig. 3b) [62]. The results suggest that, in certain cases, initially filled pores (ionophilic pores) may charge more slowly than initially empty pores (ionophobic pores) [63], though completely ionophobic pores have not yet been realized experimentally. A similar hypothesis has been revealed in the activated carbon based on *in situ* NMR (Fig. 3c) [59], in which it was found that confinement in the micropores decreases the *D_δ_* of ions by over two orders compared with bulk electrolyte. These results indicate that local heterogeneities (e.g. defects) in realistic carbon channels results in a significant difference in ionic kinetics when compared to the situation for simulation, which means that the space for improved performance if the high *D_δ_* can be well maintained. On the other hand, N sites in ionic channels (corresponding to ionophilic self-populating channels), might also raise the kinetic barriers in charging of supercapacitors. Obviously, a more fundamental understanding of heteroatom effects on the ion response is yet to be revealed, especially for those doped carbons designed for non-aqueous electrolytes [64].

In batteries, curvature and doping may efficiently increase the adsorption of Li^+^ on a carbon surface, which can be ascribed to the increased electron transfer on the defect/electrolyte interface, and sequentially promoting ion transfer [18,48]. A high capacity of 2000 mAh/g was demonstrated in carbon nanotubes with a defected N configuration [65], via which Li^+^ can diffuse easily so as to occupy more interwall nano-space as host regions, demonstrating the potential of doped carbon in high-capacity LIBs (Fig. 3d). But the effect of defects is not straightforward: Li^+^ diffusion perpendicular to the basal plane of carbon might be facilitated by defects, whereas parallel diffusion could be limited due to the steric hindrance that derives from abundant Li^+^ aggregated in the defect sites, as illustrated in Fig. 3e [66]. It is worth noting that defects may induce undesirable side reactions in batteries, which are specifically remarkable for carbons with high SSAs, leading to increased reactivity with the electrolyte and thus highly irreversible capacity at the first cy77cle and uncontrollable solid electrolyte interphase (SEI) [21,32,67].

### GRAPHENE STACKING FOR EFFICIENT AND FUNCTIONALIZED IONIC CHANNELS

Compared to complex 3D porous carbons, graphene stacking would provide relatively simple 2D ionic channels for the fundamental understanding of ionic adsorption/transport (Fig. 4a) [68], and graphene with designed defects allows an

experimental mimicking of the charge separation at the electrolyte/ultrahigh SSA carbon interface, while less affected by the tortuous pore structure in traditional activated carbon materials. Beyond that, the interlayer distance (*d*) in the stacking can be well adjusted, e.g. by exchanging with a miscible mixture of volatile and non-volatile liquids (typically, sulfuric acid), in a range from sub-nanometer to few nanometers under capillary compression (Fig. 4b) [69]. The resulting ionic channels demonstrated a low relaxation time of 0.73 s even for a film with a high packing density of 1.33 g/cm^3^ when acting as an electrode for supercapacitors [69]. Encouraged by this, planar devices have been developed to explore the full potential use of the interspace of graphene [70–72]. A stacking of multilayered CVD graphene has led to an average interlayer channel of ∼1.2 nm [73], demonstrating an excellent rectification performance with a resistance capacitance (RC) time constant (τ_0_ = RC) of 0.54 ms from the interdigital microelectrodes made by direct laser writing. In addition, the transport behavior of ions in such confined channels could also be tuned by the surface potential [74]. As shown in Fig. 4c and d, the regulated diffusion rates of K^+^ were anomalously enhanced by 4–7 times within ±0.5 V in a sub-2 nm ionic channel depending on the potential sign; this has been suggested to be the effect of strong ion–ion correlations in the interfacial electrical double layer (EDL) based on Poisson–Nernst–Planck (PNP) modeling. Graphene stacking can also be used to study the charging process based on atomic ionic diffusion and asymmetric ionic transport. For example, Wei [75] and Xia [76] *et al*. demonstrated that the water molecules confined within the nanochannels of reduced graphene oxide (rGO) and graphene oxide (GO) become more ordered under an external electrical field, leading to a local redistribution and separation of charges. Further studies are thus necessary to explore how the ion population and ion selectivity vary during electrochemical charging [77], among specific 2D channels with or without surface modification.

In batteries, on the other hand, the diffusion coefficient (*D*) of Li ions within the graphene bilayered channel (BLG) can be as high as 7 × 10^−5^ cm^2^/s during lithiation at a low potential of 0.05 V (versus Li/Li^+^), which is much faster than in graphite [78]. In this regard, it would be highly interesting to study whether similar diffusion phenomena remain in real 3D yet planar channels based on carbon electrode of graphene stacking and assembling, in which charging processes and ionic kinetics shall be different from ideal slit channels due to the increased trap states, as mentioned above. Recently, Ji *et al.* demonstrated that the C*R*C-stacking manner (*R* is the internal Li layer between carbons) goes through several stoichiometric LiC*_x_* phases before eventually achieving the LiC_6_ composition, and no fundamental difference between 3D bilayer graphene foam and graphite electrodes has been found yet in terms of Li-configuration and Li-kinetics behavior [79]. However, the case changes for a larger amount of Li-intercalation. *In situ* transmission electron microscopy (TEM) has been applied to a device with LiTFSI solid polymer electrolyte and bilayer graphene electrode (Fig. 4e) [80]. Interestingly, the formation of a multilayer close-packed Li phase between the bilayer graphene was observed, via facile diffusion of Li^+^ by an ion-exchange mechanism, leading to Li-storage capacity far exceeding the LiC_6_ composition in graphite (Fig. 4f). This finding thus points out the key role of electronic properties and spatial structures for 2D layered channels in ion storage when compared to their bulk compounds. Recent work on Li^+^ storage on single-layer graphene (SLG) also showed the possible presence of metallic Li species embedded in SEI, which may contribute to the ultrahigh specific capacity reported in many carbon anodes (Fig. 4f) [81]. These results suggest that the atomic channels between graphene platelets may help to probe new types of intercalate ordering or ionic diffusion characters for better understanding of electrochemical energy storage, and it is therefore worth making further quantitative studies on novel electrochemical devices with Li^+^ and beyond (e.g. SIBs) [4,82] based on designed graphene-stacked structures (Fig. 4f).

### OUTLOOK

As we have seen, the ionic channels in nanocarbons considerably improve the energy storage performance, in terms of energy and power density, lifetime and stability. Looking forward, however, the design of ionic channels in novel carbons is still at the early stage, with many new phenomena to be solved. The following aspects may be considered for the further development of the topic.

(i) *Smart design*: In many methods templates are required; sequential steps for removing such templates are involved. This technique is not readily scaled up for commercial applications; the residues are also undesirable for applications. Physical or chemical etching for controllable ionic channels is still far away from rational design. One route is using better defined structures, e.g. metal–organic frameworks (MOFs) and covalent organic frameworks (COFs) with good conductivity and high SSAs. It has been reported that π-conjugated 2D layers penetrated by 1D cylindrical ion channels with ∼1.5 nm diameter have been utilized for EDLCs [83], and another 2D-conjugated aromatic polymeric crystal constructed by C–C coupling shows a distinct lamellar structure with highly uniform 1D open channels of ∼0.6 nm diameter, allowing fast and smooth Na^+^ diffusion in SIBs [84]. These may help to develop bottom-up synthesis of carbon crystal (e.g. periodic *sp*^2^ carbons) as active electrode. Overall, oriented mesoporous channels (ionophobic or hydrophilic), along with micropores in the channel walls, may facilitate the ion penetration and diffusion pathways in both the in-plane and vertical directions, which is very promising for energy applications with practical levels.

(ii) *Identifying correlations*: For an ionic channel, it is critical to understand the basic and key parameters such as the efficient size of a channel, ion diffusion coefficient, wettability of electrolyte and ion selectivity under standard protocol. For example, when we try to specify the ‘transport/diffusion efficiency’ for designed ionic channels, typically, the time constant (τ_0_), as well as the diffusion coefficient (*D*) in supercapacitors and batteries, the obtained values are often dependent on the thickness of active electrodes and need to be precisely decided [85]. The exciting results from various DFT and MD studies indicate that carbon–ion interaction and ion–ion packing may take effect together in a nanoconfined channel, but these effects are yet to be quantized experimentally. Thus, it is highly expected to develop advanced *in situ* or *operando* characterizations to define descriptors such as ‘*D*’ and ion–carbon interaction energy for ionic channels in electrochemical processes; NMR seems to be a proper ionic diffusion probe in terms of spatial resolution [59]. In particular, benefiting from quasi-periodic structures, ordered graphene stacking and carbon crystal may help to understand the diffusion features of the different confined ions. If so, it would be possible to control the charging kinetics and thus further improve the capacity.

(iii) *Mimicking nature*: Ionic channels in nature regulate vital functions in life processes and specific ion-transport microstructures in living systems have become inspirations for sustainable development applications [86,87]. Experiments and simulations have shown the ultrafast and selective permeation of water and ions through interstitial nanocapillaries of graphene in symmetric channels, by reducing the fluidic resistance under the help of confined and functionalized walls (typically, with oxygen functional groups) [74,88]. Besides, a net ion diffusion current may be generated from asymmetrically charged nanochannels, which are the foundation of functional nanofluidic devices [89]. Asymmetric ion transport arises from symmetry breakdown in the structure of fluidic devices (e.g. mixed-dimensional heterojunctions) [90], or in the local fluidic environment (e.g. heterogeneous carbon nanochannels) [91], in which a preferential direction for ion transport and ion selectivity can be controlled in the charged channels. Typically, the output power density of a charged graphene oxide film (GOM) has approached 0.77 W m^−2^ driven by a chemical concentration gradient [92]. In such a situation, the negatively charged and positively charged GOM could harvest energy from the unipolar cation and anion flow in the layered channels, respectively. Thus, a superposed membrane potential is generated by packing the oppositely charged channels in parallel, which has also been utilized to fabricate nanofluidic energy-conversion devices based on mesoporous carbons [93]. Angstrom-scale channels with atomically flat walls have also been reported in graphene and other 2D nanofluidic devices. In such cases, the controllable capillaries with dimensions approaching the size of solvated ions have clearly demonstrated the role of steric effects, evidenced by the non-linear mobilities of ions due to distortions of confined hydration shells [94]. An applied electric force, on the other hand, further increased the measured pressure-driven ionic transport in angstrom-scale confinement by up to 20 times, which may offer new routes to control molecular and ion transport [95]. With the improvement in engineering and scaled-up production of atomically precise channels and pores, these systems may be combined in energy storage devices, for multi-functional design of energy applications.
